# Interaction of hemoglobin, transfusion, and acute kidney injury in patients undergoing cardiopulmonary bypass: a group-based trajectory analysis

**DOI:** 10.1080/0886022X.2022.2108840

**Published:** 2022-08-10

**Authors:** Lingyong Cao, Weizhe Ru, Caibao Hu, Yanfei Shen

**Affiliations:** a Department of Internal Medicine, Zhejiang Chinese Medical University, Hangzhou, China; b Department of Oncology, Cixi People’s Hospital, Cixi, China; c Department of Intensive Care, Zhejiang Hospital, Hangzhou, China

**Keywords:** Anemia, acute kidney injury, cardiopulmonary bypass, transfusion, trajectory

## Abstract

Anemia is a risk factor for acute kidney injury (AKI) following cardiopulmonary bypass (CPB). Whether red blood cell (RBC) transfusion-enhanced hemoglobin levels contribute to low AKI rates remains unclear. We investigated the interaction between hemoglobin, RBC transfusion, and AKI after CPB. Hemoglobin trajectories within 72 h were analyzed using group-based trajectory analysis. Multivariable logistic analysis and inverse probability-weighted regression were adopted to evaluate the associations between hemoglobin and AKI in RBC and non-RBC transfusion subgroups. We analyzed 6226 patients’ data. In the transfusion subgroup, three hemoglobin trajectories were identified. The AKI incidence was lowest in the trajectory with the lowest hemoglobin level (trajectory 1, less transfusion), and it was comparable in trajectories 2 and 3 (20.7% vs. 32.7% vs. 29.4%, *p* < 0.001, respectively). In four logistic models, the odds ratio for AKI with trajectory 1 as the reference ranged from 1.44 to 1.85 for trajectory 2 (*p* < 0.001) and 1.45 to 1.66 for trajectory 3 (*p* < 0.050). The average treatment effect on AKI was 5.6% (*p* = 0.009) for trajectory 2 and 7.5% (*p* = 0.041) for trajectory 3, with trajectory 1 as the reference. In the non-RBC transfusion subgroup, three approximately linear hemoglobin trajectories (9, 10, and 12 g/dL) were observed; however, both the crude and adjusted AKI incidence were similar within the three trajectories. In patients undergoing CPB, hemoglobin level >9 g/dL was not associated with decreased AKI incidence in the subgroup without RBC transfusion. However, in patients with RBC transfusion, maintaining hemoglobin level >9 g/dL by RBC transfusion was associated with increased AKI incidence.

## Graphical Abstract

**Figure:**
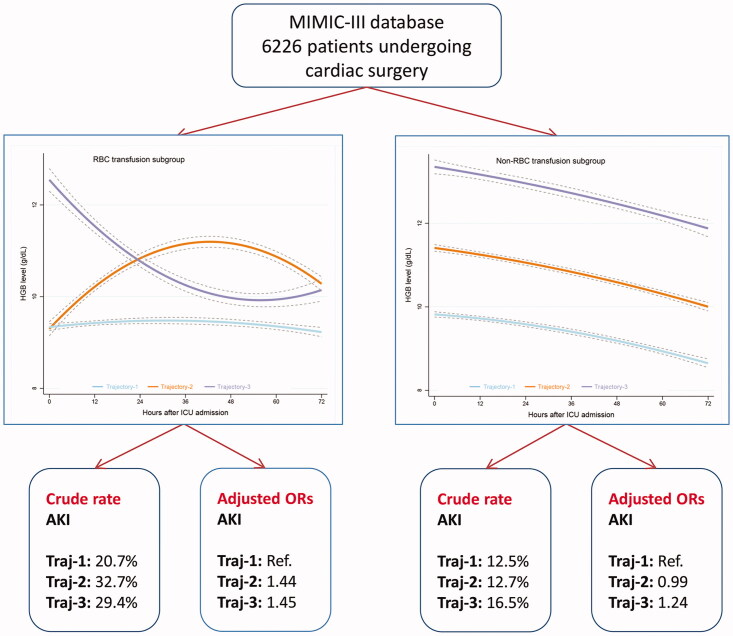

## Introduction

Acute kidney injury (AKI) is a common but severe complication of cardiac surgery [1], with a reported incidence ranging from 15% to 50% [2,3]. Development of AKI is independently associated with an increased risk of morbidity and mortality [4–6]. Considering that there is currently no effective treatment for AKI [7], identifying the modifiable risk factors for AKI is critical to minimizing its incidence [8].

Anemia is common in patients undergoing cardiac surgery [9–12]. Previous studies have found that preoperative anemia is independently associated with adverse outcomes in patients undergoing cardiac surgery. For example, in an observational study of 1214 patients undergoing coronary artery bypass grafting, preoperative anemia (≤12 g/dL) was associated with an increased incidence of AKI postoperatively [12]. Another study of 1360 patients [13] reported a significant association between a perioperative nadir hemoglobin level of ≤8 g/dL and adverse outcomes after cardiac surgery, including AKI.

However, in most of the previous studies, the definitions of anemia were inconsistent and the results were based on a single static hemoglobin value. Lack of consideration of longitudinal dynamic changes in the hemoglobin level may increase the risk of bias when assessing the association between hemoglobin and clinical outcomes. Furthermore, red blood cell (RBC) transfusion is a common strategy for treating anemia. However, a growing body of evidence [14–16] suggests an association between RBC transfusion and an increased risk of AKI in patients after cardiac surgery. Therefore, the potential benefits of high hemoglobin levels and the potential hazards of transfusion make clinical decision-making difficult. The optimal balance between the perioperative hemoglobin level and transfusion threshold remains unclear.

This study aimed to explore the interaction of longitudinal hemoglobin level, RBC transfusion, and incidence of AKI in patients undergoing cardiopulmonary bypass (CPB) using a group-based trajectory method.

## Materials and methods

### Data source and ethics approval

The data used in this study were extracted from the Medical Information Mart for Intensive Care (MIMIC) III database [17] published by the Massachusetts Institute of Technology (MIT) Computational Physiology Laboratory. Yanfei Shen had access to this database (certification number: 1564657) and was responsible for data extraction. The analysis of this data was approved by the institutional review boards of MIT and the Beth Israel Deaconess Medical Center. The requirement for informed consent was waived by the institutional review boards of MIT and the Beth Israel Deaconess Medical Center (https://www.ncbi.nlm.nih.gov/pmc/articles/PMC4878278/).

### Cohort definition

Patients who had undergone CPB were screened for inclusion in the analysis. Patients were included in the study if they were aged >18 years and had a length of hospital stay of >72 h. Patients with no hemoglobin data were excluded. For the purposes of analysis, patients were divided into two subgroups according to whether or not they received an RBC transfusion, which was defined as any dose of RBCs transfused within 72 h after intensive care unit (ICU) admission.

### Data extraction

Clinical and laboratory data, including patient demographics, comorbidities, and laboratory findings, were extracted from the MIMIC III database. Data on hemoglobin levels within 72 h after admission to the ICU were collected for trajectory modeling. Data on the daily RBC transfusion volume within 72 h were extracted. In patients with multiple ICU admissions, only the first ICU admission was included in the analysis. Variables with >20% of values missing were not imputed. For continuous variables with < 20% of values missing, the missing values were replaced with the mean or median value [18].

### Primary outcome

The primary outcome was the incidence of AKI, which was defined according to the creatinine-based Kidney Disease Improving Global Outcome criteria, without urine output [19,20]. According to the current guidelines, AKI is defined according to urine output and the change in serum creatinine levels. However, in the current study, urine output was not used in the definition of AKI due to the lack of hourly urine volume data. The creatinine value closest to surgery (before ICU admission) was defined as the baseline value [21,22]. AKI was defined as a 1.5-fold increase in the serum creatinine level after ICU admission relative to the baseline level. The severity of AKI was graded according to the Kidney Disease Improving Global Outcome guidelines.

### Group-based trajectory models

Group-based trajectory modeling [23] was used to identify hemoglobin trajectories by mapping dynamic changes in the hemoglobin value within 72 h of ICU admission. The model was constructed as follows: first, the number of trajectories was determined according to the Bayesian information criterion; second, the complexity of the model was determined on the basis of the logarithm of the Bayes factor [2log_e_(B_10_)]; and third, the average posterior probability (AvePP) was calculated to evaluate the posterior probability of each individual being assigned to the corresponding hemoglobin trajectory, with an acceptable value of 0.70.

### Statistical analysis

Normally distributed continuous variables were compared using the Student’s *t*-test and reported as the mean (standard deviation). Non-normally distributed continuous variables are reported as the median (25–75th percentiles) and were compared using the Wilcoxon rank-sum test. Categorical variables were compared using the chi-squared test. Multivariable logistic regression was used to adjust for confounding factors when exploring the association between hemoglobin trajectories and the risk of AKI. Multivariable logistic models were developed by including variables with a *p*-value of <0.20 in univariate analysis and using the stepwise backward method to select covariables. The variance inflation factor was used to test for multicollinearity, with a value of ≥5 indicating significant multicollinearity. Inverse probability-weighted regression adjustment was also used to control for confounding to obtain robust estimates. Variables within the hemoglobin trajectories that were imbalanced were included in a hemoglobin trajectory assignment (propensity score) model, and the estimated average treatment effect within each hemoglobin trajectory category was investigated in the final model. All statistical analyses were performed using Stata release 11.2 (StataCorp LLC, College Station, TX, USA). All tests were two-sided and a p-value of 0.05 was used as the threshold for statistical significance.

## Results

### Comparisons between AKI and non-AKI groups

A total of 6226 patients were included in the analysis. Detailed comparisons between the AKI and non-AKI groups are shown in Table 1. Compared with the non-AKI group, the initial (9.9 [1.7] vs. 9.8 [1.9]) and maximum (11.3 [1.3] vs. 11.6 [1.3])) hemoglobin levels within 72 h of ICU admission were similar in the AKI group, although the differences in hemoglobin were statistically significant. Vasopressors (40.3% vs. 25.9%, *p* < 0.001) and RBC transfusion (59.3% vs. 40.5%, *p* < 0.001) were significantly more likely to be used in the AKI group than in the non-AKI group.

**Table 1.** Comparisons of baseline characteristics between AKI and non-AKI groups.

Demographics	Non-AKI group (*n* = 5110)	AKI group (*n* = 1116)	All (*n* = 6226)	*p*
Age (years)	66.0 ± 11.2	70.0 ± 11.9	66.7 ± 12.3	<0.001
Male [*n* (%)]	3631 (71.0)	660 (59.1)	4291 (68.9)	<0.001
Hypertension [*n* (%)]	3145 (61.5)	626 (56.0)	3771 (60.5)	0.001
Diabetes mellitus [*n* (%)]	1491 (29.1)	361 (32.3)	1852 (30.2)	0.036
Laboratory indexes				
Initial WBC count (10^9^/L)	12.6 ± 5.0	13.0 ± 6.1	12.7 ± 5.2	0.024
Maximum WBC count (10^9^/L)	15.3 ± 5.4	18.0 ± 7.7	15.8 ± 6.0	<0.001
Initial hemoglobin level (g/dl)	9.9 ± 1.7	9.8 ± 1.9	9.9 ± 1.7	<0.001
Maximum hemoglobin level (g/dl)	11.3 ± 1.3	11.6 ± 1.3	11.3 ± 1.3	<0.001
Initial platelet count (10^9^/L)	166.3 ± 62.4	164.8 ± 72.0	166.0 ± 64.3	0.464
Minimum platelet count (10^9^/L)	136.0 ± 51.6	117.9 ± 55.8	132.7 ± 52.8	<0.001
Baseline serum creatinine (mg/dl)	1.1 ± 0.9	0.9 ± 0.5	1.0 ± 0.8	<0.001
Maximum serum creatinine (mg/dl)	1.1 ± 0.7	1.7 ± 1.3	1.2 ± 0.9	<0.001
Coronary angiography [*n* (%)]	1965 (38.4)	388 (34.7)	2253 (36.8)	0.276
Intra-aortic balloon pump [*n* (%)]	361 (7.0)	139 (12.4)	500 (8.0)	<0.001
Operation on valves [*n* (%)]*	1892 (37.0)	541 (48.4)	2433 (39.1)	<0.001
Coronary artery bypass grafting [*n* (%)]	3649 (71.4)	739 (66.2)	4388 (70.4)	0.001
Clinical evaluation				
Vasopressor-use [*n* (%)]	1324 (25.9)	450 (40.3)	1774 (28.4)	<0.001
RBC transfusion [*n* (%)]	2071 (40.5)	662 (59.3)	2733 (43.8)	<0.001
SOFA at ICU admission [median (IQR)]	4 (3 − 6)	5 (3 − 7)	4.5 (3 − 6)	<0.001
Maximum SOFA [median (IQR)]	9 (7 − 11)	11 (8 − 13)	9 (7 − 11)	<0.001
Hospital LOS (days)	7.0 (5.2 − 10.0)	10.9 (7.1 − 17.3)	7.4 (5.3 − 11.0)	<0.001
AKI I [*n* (%)]	0	740 (66.3)	740 (11.8)	
AKI II [*n* (%)]	0	247 (22.1)	247 (3.9)	
AKI III [*n* (%)]	0	129 (11.5)	129 (2.0)	

AKI: acute kidney injury; ICU: intensive care unit; IQR: interquartile range; LOS: length of stay; RBC: red blood cell; SOFA: Sequential Organ Failure Assessment; WBC: white blood cell.

*Operation on valves including all operations on mitral, aortic, tricuspid and pulmonary valves.

### Hemoglobin trajectories in the RBC transfusion subgroup

**Figure 1.**
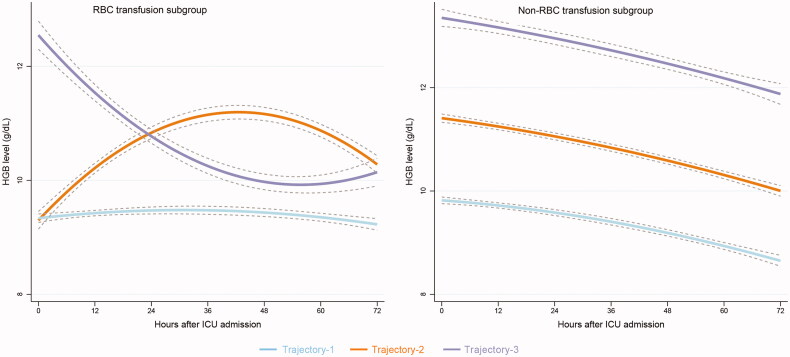
Hemoglobin-based trajectories in patients with and without red blood cell transfusion. Left panel: patients with RBC transfusion. Trajectory-1 (*n* = 1880), a HGB trajectory approximately 9 g/dL; Trajectory-2 (*n* = 602), HGB increased from 9 to 11 g/dL within 36 h and then decreased gradually to 10 g/dL; Trajectory-3 (*n* = 251), HGB rapidly decreased from 12 to 10 g/dL within 36 h and was maintained at approximately 10 g/dL. Right panel: patients without RBC transfusion. Trajectory-1 (n = 1672), HGB gradually decreased from 10 to 9 g/dL; Trajectory-2 (n = 1518), HGB gradually decreased from 11 to 10 g/dL; Trajectory-3 (*n* = 303), HGB gradually decreased from 13 to 12 g/dL. HGB: hemoglobin; ICU: intensive care unit; RBC: red blood cell.

Total of 27782 hemoglobin measurements were extracted. Different hemoglobin trajectories were found in patients with and without RBC transfusion according to the Bayesian information criterion and tests for statistical significance. In the RBC transfusion subgroup, three hemoglobin trajectories were identified (Figure 1): trajectory 1 (AvePP 0.88, *n* = 1880), in which hemoglobin was maintained at approximately 9 g/dL; trajectory 2 (AvePP 0.79, *n* = 602), in which hemoglobin increased from 9 g/dL to 11 g/dL within 36 h and then decreased gradually to 10 g/dL; and trajectory 3 (AvePP 0.82, *n* = 251), in which hemoglobin decreased rapidly from 12 g/dL to 10 g/dL within 36 h and was then maintained at approximately 10 g/dL. According to the logarithm of the Bayes factor, each trajectory was described by a unique quadratic equation in which hemoglobin was a function of time.

### Comparisons within hemoglobin trajectories in the RBC transfusion subgroup

The crude outcome comparisons among three trajectories in the RBC transfusion subgroup are shown in Table 2. Compared with patients with trajectory 1, those with trajectories 2 and 3 were more likely to be treated with a vasopressor (37.9%, 50.6%, and. 53.7%, respectively, *p* < 0.001), have a longer hospital stay, and have a higher incidence of AKI (20.7%, 32.7%, and 29.4%, respectively, *p* < 0.001). On day 1, patients with trajectory 2 received a higher RBC transfusion volume that those with trajectories 1 and 3 (949 [781] mL, 724 [532] mL, and 710 [529] mL, respectively, *p* < 0.001). On days 2 and 3, the difference in the RBC transfusion volume became small among the three trajectories.

**Table 2.** Crude comparisons within HGB trajectories in RBC and non-RBC transfusion subgroups.

	RBC transfusion subgroup				Non-RBC transfusion subgroup			
	Trajectory 1 (*n* = 1880)	Trajectory 2 (*n* = 602)	Trajectory 3 (*n* = 251)	*p*	Trajectory 1 (*n* = 1672)	Trajectory 2 (*n* = 1518)	Trajectory 3 (*n* = 303)	*p*
SOFA at ICU admission [median (IQR)]	5 (4–7)	6 (4–8)	4 (2–7)	<0.001	4 (3–6)	4 (3–6)	3 (1–5)	<0.001
Maximum SOFA [median (IQR)]	9 (8–11)	11 (9–13)	11 (9 − 13)	<0.001	8 (6–10)	8 (7–10)	8 (6–10)	0.006
Vasopressor-use [*n* (%)]	713 (37.9)	305 (50.6)	135 (53.7)	<0.001	297 (17.7)	278 (18.3)	46 (15.1)	0.428
Coronary angiography [*n* (%)]	722 (38.4)	219 (36.3)	135 (53.7)	<0.001	546 (32.6)	499 (32.8)	132 (43.5)	0.001
Intra-aortic balloon pump [*n* (%)]	140 (7.4)	76 (12.6)	96 (38.2)	<0.001	44 (2.6)	101 (6.6)	43 (14.1)	<0.001
Operation on valves [*n* (%)]*	859 (45.6)	314 (52.1)	71 (28.2)	<0.001	610 (36.4)	494 (32.5)	85 (28.0)	0.005
Coronary artery bypass grafting [*n* (%)]	1329 (70.6)	396 (65.7)	206 (82.0)	<0.001	1160 (69.3)	1077 (70.9)	220 (72.6)	0.415
Initial cardiac output (L/min)	4.9 ± 1.4 (*n* = 561)	4.2 ± 1.3 (*n* = 187)	4.5 ± 1.2 (*n* = 61)	<0.001	5.3 ± 1.5 (*n* = 485)	5.4 ± 1.4 (*n* = 456)	5.6 ± 1.3 (*n* = 96)	0.046
Hospital LOS (days)	8.3 (6.1 − 12.2)	10.1 (6.8 − 16.4)	9.7 (6.8 − 14.1)	<0.001	6.8 (5.1 − 9.1)	6.5 (5.0 − 9.1)	7.9 (5.5 − 12.7)	0.001
AKI incidence [*n* (%)]	391 (20.7)	197 (32.7)	74 (29.4)	<0.001	210 (12.5)	194 (12.7)	50 (16.5)	0.162
AKI I [n (%)]	255 (13.5)	101 (16.7)	47 (18.7)		160 (9.5)	146 (9.6)	31 (10.2)	
AKI II [*n* (%)]	96 (5.1)	61(10.1)	15 (5.9)		33 (1.9)	29 (1.9)	13 (4.2)	
AKI III [*n* (%)]	40 (2.1)	35 (5.8)	12 (4.7)		17 (1.0)	19 (1.2)	6 (1.9)	
RBC transfusion on Day 1 (ml)	724 ± 532	949 ± 781	710 ± 529	<0.001				
RBC transfusion on Day 2 (ml)	536 ± 321	632 ± 545	684 ± 420	<0.001				
RBC transfusion on Day 3 (ml)	542 ± 416	521 ± 293	576 ± 311	0.564				
Volume of drainage fluid (ml/72h)	1294 ± 987	1460 ± 1140	1454 ± 1079	<0.001	762 ± 397	794 ± 542	703 ± 374	0.015

AKI: acute kidney injury; ICU: intensive care unit; IQR: interquartile range; LOS: length of stay; RBC: red blood cell; SOFA: Sequential Organ Failure Assessment.

*Operation on valves including all operations on mitral, aortic, tricuspid and pulmonary valves.

The association between hemoglobin (HGB) trajectories and AKI incidence was also investigated in four logistic regression models, including different adjusted variables (Table 3, Table S1). The odds ratio (OR) for AKI with trajectory 1 as the reference ranged from 1.44 to 1.85 for trajectory 2 (*p* < 0.001) and 1.45 to 1.66 for trajectory 3 (*p* < 0.050). Similarly, in the adjusted inverse probability-weighted regression model (Table 4), the average reduced incidence of AKI attributable to the hemoglobin trajectory relative to trajectory 1 was 5.6% (*p* = 0.009) for trajectory 2 and 7.5% (*p* = 0.041) for trajectory 3.

**Table 3.** Association between hemoglobin trajectory and risk of acute kidney injury in different logistic regression models.

RBC transfusion subgroup (*n* = 2733)			Non-RBC transfusion subgroup (*n* = 3493)		
Variables	Adjusted OR (95% CI)	*p*	Variables	Adjusted (95% CI)	*p*
Model 1			Model 1		
Traj-1	Ref.	–	Traj-1	Ref.	–
Traj-2	1.85 (1.51–2.27)	<0.001	Traj-2	1.02 (0.82–1.25)	0.852
Traj-3	1.59 (1.18–2.13)	0.002	Traj-3	1.37 (0.98–1.92)	0.063
Model 1			Model 1		
Traj-1	Ref.	–	Traj-1	Ref.	–
Traj-2	1.77 (1.44–2.18)	<0.001	Traj-2	1.03 (0.83–1.27)	0.762
Traj-3	1.66 (1.23–2.24)	0.001	Traj-3	1.15 (0.96–1.90)	0.080
Model 1			Model 1		
Traj-1	Ref.	–	Traj-1	Ref.	–
Traj-2	1.70 (1.37 − 2.10)	<0.001	Traj-2	1.00 (0.80–1.22)	1.000
Traj-3	1.54 (1.14–2.10)	0.005	Traj-3	1.28 (0.90–1.80)	0.160
Model 1			Model 1		
Traj-1	Ref.	–	Traj-1	Ref.	–
Traj-2	1.44 (1.15–1.80)	0.001	Traj-2	0.99 (0.79–1.23)	0.931
Traj-3	1.45 (1.04–2.01)	0.026	Traj-3	1.24 (0.86–1.79)	0.236

Note. Model 1: crude OR; Model 2: Model 1 + hypertension, diabetes, COPD, and sepsis; Model 3: Model 2 + white blood cell, platelet, serum sodium, calcium, and baseline creatinine level; Model 4: Model 3 + vasopressor use, fluid balance, coronary angiography, intra-aortic balloon pump, and SOFA score.

CI: confidence interval; COPD: chronic obstructive pulmonary disease; OR: odds ratio; SOFA: Sequential Organ Failure Assessment.

**Table 4.** Estimated average treatment effects on AKI incidence in multivariable inverse probability-weighted regression models.

RBC transfusion subgroup (*n* = 2733)			Non-RBC transfusion subgroup (*n* = 3493)		
Variables	Adjusted ATE (95% CI)	*p*	Variables	Adjusted ATE (95% CI)	*p*
Traj-1	Ref.	–	Traj-1	Ref.	–
Traj-2 vs. Traj-1	0.056 (0.014–0.098)	0.009	Traj-2 vs. Traj-1	0.008 (-0.014–0.031)	0.477
Traj-3 vs. Traj-1	0.075 (0.003–0.147)	0.041	Traj-3 vs. Traj-1	0.033 (-0.014–0.081)	0.170

AKI: acute kidney injury; ATE: average treatment effect; CI: confidence interval; HGB: hemoglobin; RBC: red blood cell; Traj: trajectory.

### Hemoglobin trajectories in the Non-RBC transfusion subgroup

In the non-RBC transfusion subgroup, three hemoglobin trajectories were identified (Figure 1): trajectory 1 (AvePP 0.9, *n* = 1672), in which hemoglobin gradually decreased from 10 to 9 g/dL; trajectory 2 (AvePP 0.8, *n* = 1518), in which hemoglobin gradually decreased from 11 to 10 g/dL; and trajectory 3 (AvePP 0.9, *n* = 303), in which hemoglobin gradually decreased from 13 to 12 g/dL.

### Comparisons within hemoglobin trajectories in the Non-RBC transfusion subgroup

The crude outcome comparisons among the three trajectories are shown in Table 2. The incidence of vasopressor use (17.7%, 18.3%, and 15.1% for trajectories 1, 2, and 3, respectively, *p* = 0.428) and AKI (12.5%. 12.7%, and 16.5%, respectively, *p* = 0.162) were similar between the three trajectories. In the four logistic regression models (Table 3, Table S1), the OR for AKI ranged from 0.99 to 1.03 for trajectory 2 (*p* > 0.050) and 1.15 to 1.37 for trajectory 3 (*p* > 0.050), relative to trajectory 1. Similarly, in the adjusted inverse probability-weighted regression model (Table 4), the average treatment effect on the incidence of AKI did not differ significantly according to the hemoglobin trajectory.

## Discussion

The strength of the current study, in comparison with previous studies, is that we used longitudinal hemoglobin data instead of a static hemoglobin value to evaluate the association between hemoglobin level and the incidence of AKI in patients undergoing CPB, while also taking into account the impact of RBC transfusion. The major finding is that in patients without RBC transfusion, high hemoglobin levels (>9 g/L) were not associated with a decreased incidence of AKI. However, in patients who received RBC transfusion, high hemoglobin levels (trajectories 2 and 3) were associated with an increased incidence of AKI. These findings were confirmed in the inverse probability-weighted regression models.

Perioperative anemia is common in patients undergoing CPB. Several studies have investigated the association between perioperative anemia and adverse postoperative outcomes in these patients. One study [24] that included 375 patients undergoing elective cardiac surgery found that low intraoperative hemoglobin during CPB was associated with a high postoperative incidence of AKI. Another multicenter study [10] that included 70 institutions showed that a low preoperative hemoglobin level was an independent predictor of poor non-cardiac outcomes, including AKI. A study of 1360 patients with CPB [13] found that the nadir hemoglobin, but not preoperative anemia, was an independent risk factor for an adverse outcome.

One major issue of the previous studies is that the definitions of anemia varied greatly, such as preoperative hemoglobin level of ≤12 g/dL [12], a nadir hemoglobin level of ≤8 g/dL [13], and a hematocrit of <30% [25]. The inconsistency of various anemia definitions increases the difficulty of the clinical application of these studies. In addition, most previous definitions of anemia were based on a single static hemoglobin value, which failed to reflect the dynamic changes in hemoglobin status in clinical practice. For instance, in our study, we found that the dynamic hemoglobin level within 72 h of ICU admission differed significantly from the initial hemoglobin level, especially in patients who received RBC transfusions. This may be one reason for these inconsistent findings [12,13,26].

Using group-based trajectory modeling, we identified three hemoglobin trajectories based on longitudinal hemoglobin data in patients without RBC transfusion. A gradually decreasing smooth trend of hemoglobin levels was observed in all three trajectories. However, compared with the lowest hemoglobin trajectory (9 g/dL), trajectories with higher hemoglobin levels (10 g/L and 12 g/dL) were not associated with a decreased incidence of AKI. This finding differs from that of other studies [10,13,24] that found anemia to be associated with a poor prognosis in patients after CPB. There are several possible explanations for this inconsistency. First, most previous studies mixed patients with and without RBC transfusion. However, we noted that the hemoglobin trajectories and their relationship with AKI incidence differed significantly in the RBC and non-RBC transfusion cohorts. Therefore, mixing these patients may have introduced a degree of bias. Second, the lowest hemoglobin level in our study was 9–10 g/dL, which is higher than the lowest hemoglobin level in some of the previous studies. Another possible explanation is that even the lowest hemoglobin level (9–10 g/dL) have provides enough oxygen delivery (DO_2_) to the kidneys [27,28], and thus higher HGB level was not associated with decreased AKI incidence. However, whether a lower hemoglobin concentration (7–8 g/dL) is adequate in these patients cannot be inferred from the current study. Noteworthy, a sub-study [29] of the TRICS-III multicenter study compared a restrictive threshold for RBC transfusion (transfuse if HGB < 7.5 g/dl) with a liberal threshold (transfuse if < HGB 9.5 g/dl in the OR or ICU, or if HGB <8.5 g/dl on the ward) and found that a restrictive approach did not reduce the AKI incidence among patients undergoing cardiac surgery.

RBC transfusion is an important treatment for anemia after cardiac surgery. In clinical practice, thresholds for RBC transfusion, such as 9 g/dL [30,31] and 10 g/dL [24], have been wildly adopted. However, whether maintaining the hemoglobin level higher was beneficial in these patients remains unclear. In this study, three different hemoglobin trajectories were identified in patients who received RBC transfusion. Compared with patients with the lowest hemoglobin level (9 g/dL in trajectory 1), those with higher hemoglobin levels (trajectories 2 and 3) had a significantly higher incidence of AKI. Of note, the initial hemoglobin value was similar in trajectories 1 and 2. However, within the following 36 h, the hemoglobin level increased rapidly from 9 g/dL to 11 g/dL in trajectory 2. Meanwhile, the RBC transfusion volume was significantly greater in trajectory 2 than in trajectory 1. Based on these findings, we infer that compared with trajectory 1, the increase in hemoglobin concentration in trajectory 2 was likely to be caused by a high RBC transfusion volume, which may be a significant cause of AKI. Therefore, we conclude that maintaining hemoglobin at >9 g/dL *via* RBC transfusion may be associated with an increased risk of AKI.

This study had some limitations. First, it had a retrospective design. Although many potential confounders were considered, some factors, such as ejection fraction, intraoperative hemoglobin, duration of CPB, and emergency status, were not available in this database, which may still increase the bias risk [8]. Second, the proportion of stage 2 and 3 AKI usually accounts for 10% of the overall AKI events. In the current study, the proportion was high (33%). Whether this proportion is caused by some relative factors, such as CPB duration and complex procedures, necessitates further investigation. Third, the lowest hemoglobin level was approximately 9 g/dL in the RBC and non-RBC transfusion subgroups. Whether a lower hemoglobin concentration (7–8 g/dL) is adequate in these patients remains unclear. Forth, although the current findings were based on longitudinal hemoglobin data, a causal relationship cannot be inferred because of the observational study design. Therefore, our findings require validation in more rigorously designed studies in the future.

## Conclusions

In conclusion, a hemoglobin level of >9 g/dL was not associated with a decreased incidence of AKI in patients undergoing CPB without RBC transfusion. Furthermore, in patients who received RBC transfusion, maintaining hemoglobin at a level of >9 g/dL by RBC transfusion was associated with an increased risk of AKI. Well-designed studies that minimize selection bias are needed to determine whether there is a causal relationship between RBC transfusion and AKI in patients undergoing CPB surgery.

## Supplementary Material

Supplemental Material

## Data Availability

All the data were extracted from the international database (MIMIC-III, https://mimic.mit.edu/docs/iii/tables/), and were available upon reasonable request by contact with the corresponding author.
