# Supplementary material for: Interaction of hemoglobin, transfusion, and acute kidney injury in patients undergoing cardiopulmonary bypass: a group-based trajectory analysis
Source: Ren Fail. 2022 Aug 10;44(1):1368–75. doi: 10.1080/0886022X.2022.2108840 (PMC9373743; doi:10.1080/0886022X.2022.2108840)
Supplement: Supplemental Material [file IRNF_A_2108840_SM2586.pdf]

Table S1 Association between hemoglobin trajectory and risk of acute kidney injury in multivariable logistic regression model

**RBC transfusion subgroup (n = 2733)**			**Non-RBC transfusion subgroup (n = 3493)**		
**Variables**	Adjusted OR (95% CI)	p	**Variables**	Adjusted (95% CI)	p
**Traj-1**	Ref.	-	Traj-1	Ref.	-
**Traj-2**	1.44 (1.15 – 1.80)	0.001	Traj-2	0.99 (0.79 – 1.23)	0.931
**Traj-3**	1.45 (1.04 – 2.01)	0.026	Traj-3	1.24 (0.86 – 1.79)	0.236
**COPD**	4.60 (1.56 – 13.6)	0.006	COPD	1.15 (0.21 – 6.10)	0.867
**Hypertension**	0.68 (0.56 – 0.82)	< 0.001	Hypertension	0.97 (0.78 – 1.21)	0.821
**Diabetes**	1.24 (1.01 – 1.52)	0.035	Diabetes	1.40 (1.12 – 1.74)	0.002
**Sepsis**	1.82 (1.45 – 2.28)	< 0.001	Sepsis	3.16 (2.39 – 4.17)	< 0.001
**Initial platelet count**	1.00 (0.99 – 1.00)	0.829	Initial platelet count	0.99 (0.99 – 1.00)	0.304
**Initial WBC count**	1.01 (0.99 – 1.03)	0.096	Initial WBC count	1.01 (0.98 – 1.02)	0.648
**Initial sodium**	1.03 (1.01 – 1.06)	0.017	Initial sodium	1.04 (1.01 – 1.08)	0.021
**Initial calcium**	1.27 (1.08 – 1.49)	0.003	Initial calcium	1.15 (0.89 – 1.49)	0.274
**Baseline creatinine**	0.58 (0.48 – 0.70)	< 0.001	Baseline creatinine	0.42 (0.29 – 0.60)	< 0.001
**Vasopressor use**	1.02 (0.82 – 1.27)	0.812	Vasopressor use	1.26 (0.95 – 1.67)	0.096
**Fluid balance**	1.007 (1.005 – 1.010)	< 0.001	Fluid balance	1.009 (1.005 – 1.013)	< 0.001
**Coronary angiography**	0.62 (0.50 – 0.77)	< 0.001	Coronary angiography	1.15 (0.92 – 1.44)	0.203
**IABP**	1.38 (1.01 – 1.90)	0.043	IABP	2.34 (1.58 – 3.46)	< 0.001

**SOFA score on admission**	1.09 (1.05 – 1.14)	< 0.001	SOFA score on admission	1.01 (0.96 – 1.07)	0.604

Abbreviations: CI, confidence interval; IABP intra-aortic ballon pump; OR, odds ratio; WBC, white blood cell; SOFA, Sequential Organ Failure Assessment;
